# Case Report: Subacute combined degeneration of the spinal cord mimic accompanying adaptor protein-3B2-IgG

**DOI:** 10.3389/fimmu.2025.1598033

**Published:** 2025-09-12

**Authors:** Ying Liu, Guo-Hui Gao, Jie Lin, Chong Sun, Yan-Yin Zhao

**Affiliations:** ^1^ Department of Neurology, The First Affiliated Hospital of Shandong First Medical University & Shandong Provincial Qianfoshan Hospital., Jinan, China; ^2^ Shandong Institute of Neuroimmunology, Jinan, China; ^3^ Shandong Provincial Medicine and Health Key Laboratory of Neuroimmunology, Jinan, China; ^4^ Department of Neurology, Huashan Hospital of Fudan University, Shanghai, China; ^5^ National Center for Neurological Disorders (NCND), Shanghai, China; ^6^ Huashan Rare Disease Center, Huashan Hospital Fudan University, Shanghai, China

**Keywords:** subacute combined degeneration, spinal cord, adaptor protein-3B2-IgG, AP3B2 antibody, sensory ataxia, peripheral neuropathy

## Abstract

We here report the first case of subacute combined degeneration (SCD)-mimic accompanying adaptor protein-3B2 (AP3B2) antibody, expanding the clinical spectrum of AP3B2 antibody-associated disorders. A 55-year-old woman presented with progressive limb numbness, gait instability, and sensory ataxia over six years, unresponsive to prolonged vitamin B12 therapy. Neurological examination revealed combined posterior column, lateral column, and peripheral nerve involvement. Cervical spinal MRI demonstrated posterior column hyperintensity, while electrophysiology confirmed sensory-predominant peripheral neuropathy. Anti-AP3B2 antibodies were detected in serum (titer 1:100), with no evidence of vitamin B12 malabsorption, copper deficiency, paraneoplastic syndromes, or other immune abnormalities. Clinically resembling SCD, key discrepancies included: initial mononeuropathy multiplex/sensory neuronopathy evolving into symmetric polyneuropathy; normal vitamin B12 metabolism despite treatment resistance; absence of megaloblastic anemia. Based on AP3B2 expression in dorsal root ganglia, spinal cord, and cerebral cortex, we propose the novel entity “anti-AP3B2 antibody-associated SCD-mimic phenotype,” highlighting its distinction from classical SCD. AP3B2 antibodies likely mediate neuronal injury via CD8^+^ T-cell cytotoxicity, consistent with intracellular antigen-targeting autoimmune mechanisms. While prior AP3B2-associated cases primarily featured cerebellar ataxia or sensory ataxia, this case uniquely manifests the SCD-like triad (posterior column, pyramidal tract, and peripheral nerve damage). Clinicians should consider anti-AP3B2 antibody screening in SCD-like patients refractory to vitamin B12 therapy. Although immunotherapy responses remain limited in reported cases, early identification may optimize diagnostic and therapeutic strategies.

## Introduction

Subacute combined degeneration of the spinal cord (SCD) is a neurological degenerative disorder caused by vitamin B12 deficiency, predominantly affecting the posterior columns, lateral columns of the spinal cord, and peripheral nerves (1). Adaptor protein-3B2 (AP3B2), previously termed β-neuronal adaptin, is critical in neuronal vesicle trafficking (2). Herein, we report a case of AP3B2 antibody positivity with clinical features highly resembling SCD. We propose the term anti-AP3B2 antibody-associated SCD-mimic to characterize this novel phenotype, thereby broadening the differential diagnostic spectrum of neurological disorders.

## Case presentation

**Figure 1:**
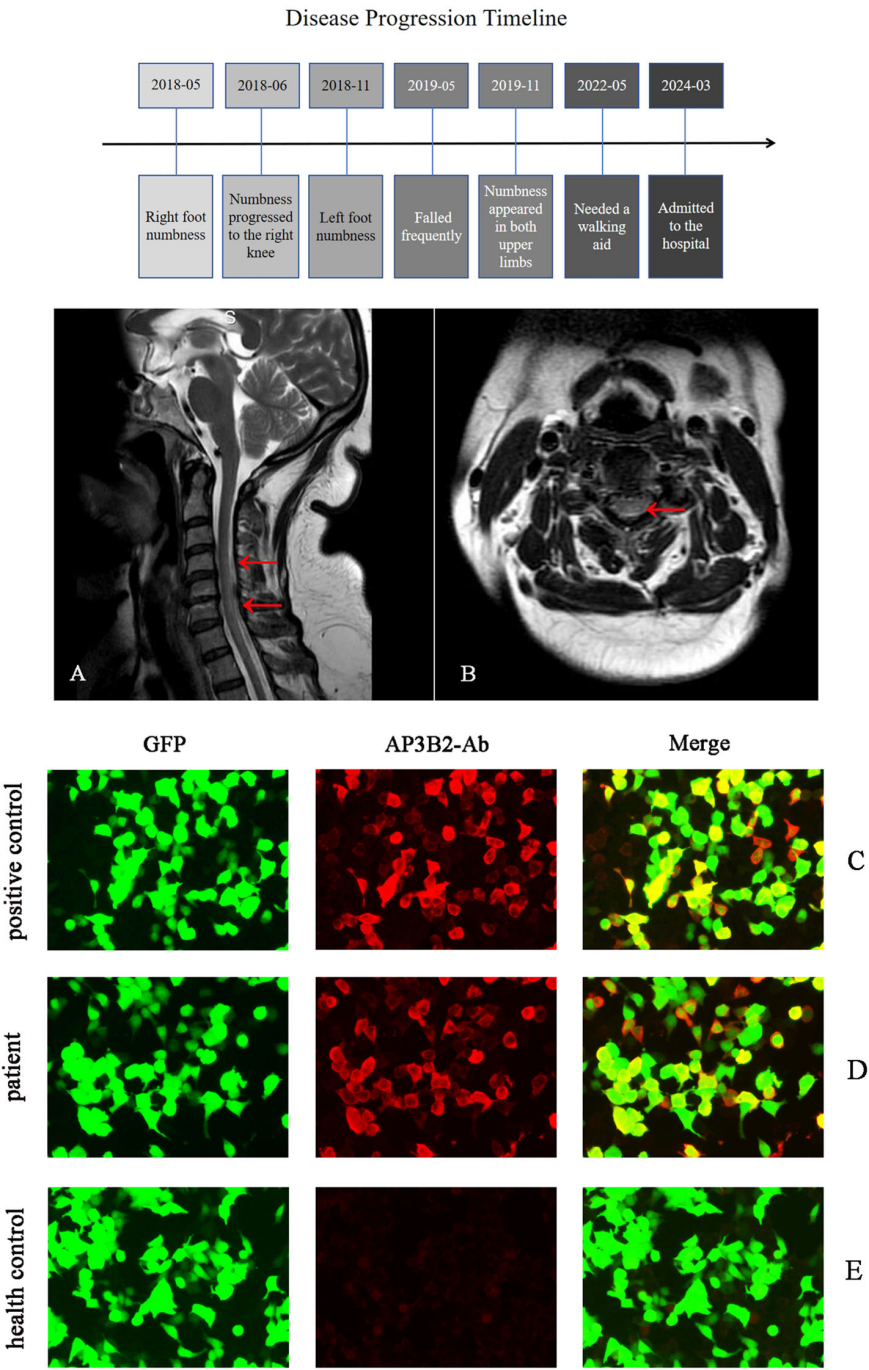
The top part shows the disease progression process of the patient. Figures **(A, B)** are the sagittal **(A)** and axial **(B)** views of the patient’s cervical spine magnetic resonance imaging, respectively. The red arrows indicate the T2 hyperintensity. The patient’s serum antibodies were detected using the cell-based assay. The positive control **(C)**, the patient’s serum **(D)**, and the serum of a healthy individual **(E)** were incubated with HEK293 cells transfected with AP3B2, simultaneously co-transfected with green fluorescent protein (GFP) as an internal reference for detection, respectively. Then, a secondary antibody labeled with red fluorescence was added. By observing the cells labeled with red fluorescence, it can be seen in **(D)** that the cells labeled with red fluorescence overlap with the green fluorescence, indicating the presence of anti-AP3B2 antibodies in the patient’s serum.

A 55-year-old female was admitted in March 2024 with a six-year history of limb numbness and gait instability. In May 2018, she developed insidious-onset numbness in her right foot, progressing to the right knee within one month. Six months later, numbness emerged in her left foot. One year post-onset, she experienced frequent falls. By 1.5 years after symptom onset, bilateral upper limb numbness developed. Four years into the disease course, she required a walking aid and avoided nighttime outings (see **Figure 1**). Notably, she lacked weakness, pain, or autonomic dysfunction. Previous SCD diagnosis at a local hospital showed no improvement with 6-month parenteral mecobalamin. The patient had no history of anemia, diabetes, alcohol abuse, gastrointestinal surgery, nitrous oxide exposure, or toxic substance contact. Family history was negative for autoimmune or hereditary neurological disorders.

Neurological examination revealed intact cranial nerves and normal muscle strength in all limbs. Vibration sense was diminished bilaterally below the anterior superior iliac spines, and pinprick sensation was reduced in the distal extremities. Romberg’s sign was positive. Tendon reflexes were absent, and Babinski sign was positive.

Routine blood tests, coagulation profile, and cerebrospinal fluid analysis revealed no abnormalities. Blood glucose, folic acid, and homocysteine levels were within normal ranges. Anti-gastric parietal cell antibodies and anti-intrinsic factor antibodies were undetectable. Elevated serum vitamin B12 levels were attributable to prolonged methylcobalamin supplementation. Serum and urine immunofixation electrophoresis were negative. Rheumatological and immunological indices revealed no abnormalities. Serological testing for syphilis and HIV returned negative. Tumor marker screening (female-specific panel) and paraneoplastic antibody assessments yielded normal results.

Cervical spinal MRI demonstrated hyperintense signals in the posterior columns (**Figures 1A, B**), while cranial MRI revealed no significant abnormalities. Electromyography findings were consistent with peripheral neuropathy predominantly affecting sensory fibers, manifesting as reduced or absent sensory nerve action potential amplitudes. Bilateral lower extremity somatosensory evoked potentials were absent.

We initially employed the tissue-based assay to screen for potential unknown antibodies in the patient’s serum. The serum was incubated with macaque cerebellar and hippocampal tissue sections for antigen-antibody binding, followed by incubation with a fluorescein-labeled anti-human IgG secondary antibody. Fluorescence microscopy revealed specific immunofluorescence signals in the cerebellar and hippocampal regions. To further confirm these findings, we performed a cell-based assay. HEK293 cells were transfected with the AP3B2 gene to express AP3B2 protein on the cell membrane. Green fluorescent protein (GFP) was co-expressed as an internal control. Twenty-four hours after transfection, the cells were incubated with diluted patient serum, followed by incubation with a red fluorescein-labeled anti-human IgG secondary antibody. Distinct red fluorescence was observed under microscopy, confirming the presence of AP3B2 antibodies at a titer of 1:100 (**Figure 1D**). In addition, we established positive controls (**Figure 1C**) and negative healthy controls (**Figure 1E**). The patient refused plasma exchange and other immunotherapies.

## Discussion

While the patient’s clinical manifestations of concurrent involvement in peripheral nerves, lateral columns, and posterior columns appeared suggestive of SCD, several inconsistencies challenge this diagnosis (1). Analysis of disease progression revealed that the pattern of sensory nerve involvement was consistent with mononeuropathy multiplex or sensory neuronopathy (SN), rather than the symmetrical polyneuropathy predominantly affecting the lower extremities characteristic of SCD. However, with disease progression, the neurological manifestations gradually evolved into polyneuropathy (2). The patient exhibited no impairments in vitamin B12 uptake, absorption, binding, or transport mechanisms, yet showed suboptimal response to prolonged vitamin B12 supplementation (3). The absence of megaloblastic anemia further contradicts classical SCD pathophysiology.

Copper deficiency myelopathy, whose clinical and radiological manifestations are nearly indistinguishable from those of SCD, is typically associated with cytopenia and low serum copper and ceruloplasmin levels (3). However, this patient had no identifiable risk factors for copper deficiency and exhibited normal serum copper levels.

AP3B2 is expressed in cerebellar Purkinje cells, dorsal root ganglia, sympathetic ganglia, spinal cord, and cerebral cortex. Neurological disorders associated with anti-AP3B2 antibodies may manifest as cerebellar ataxia, myeloneuropathy, sensory ataxia, dysautonomia, and overlapping manifestations of these conditions (4). To date, 14 cases (**Table 1**) of AP3B2 antibody-associated disorders have been documented in the literature (4–7). In our case, the triad of sensory neuropathy, pyramidal tract involvement, and posterior column impairment is attributable to AP3B2 expression in the dorsal root ganglia, spinal cord, and cerebral cortex. The convergence of these three phenotypic features mimics the clinical presentation of SCD, prompting us to term this novel entity the SCD-mimic phenotype.

**Table 1:** Clinical manifestations of anti-AP3B2 antibody-associated disorders.

Patients	Author/time	Age/ gender	Main neurological symptoms and signs	Diagnosis	Neuroimaging	Electrophysiologic findings	Treatment (improvement)	Accompanying tumor	Comorbidity	Follow-up duration
P1	Darnell et al./1991	35/F	Dysarthria, vertigo, cerebellar ataxia, rapid progress over 2 months	Acute cerebellar ataxia, pyramidal tract involvement	Normal	NA	None (stable)	None	–	30 months
P2	Honorat et al./ 2019	29/F	Balance difficulty, dysarthria, dizziness, ataxia, tremor, vertigo	Cerebellar ataxia	MRI brain: cerebellar atrophy prominent in vermis	NA	NA		Hypothyroidism	7 months
P3		58/F	Paresthesia, impaired balance and gait, bilateral lower extremity weakness, urinary urgency and incontinence, constipation	Myeloneuropathy, dysautonomia	MRI brain: normal; MRI cord: cord atrophy, dorsal LETM	Severe polyradiculopathy affecting thoracic and lumbosacral segments	CS, PE, IVIG, CTX (ineffective)	Renal cell carcinoma		60 months (died)
P4		24/F	sensory ataxia, weakness, gastroparesis	Sensory neuropathy, dysautonomia		sensory neuropathy	CS, IVIG (ineffective)			36 months
P5		30/F	Right hand numbness, paresthesiae, dizziness, vertigo	Myeloneuropathy	MRI cord: tractopathy (T2 signal in posterior columns)	diffuse large fiber sensory neuropathy	IVIG, RTX, MMF (ineffective)		Sjogren syndrome	94 months
P6		44/M	Myalgia, neuropathy	Sensory neuropathy	NA	NA	NA	Remote B cell lymphoma		30 months (died)
P7		40/F	Gait disturbance	Myeloneuropathy	MRI cord: spinal cord atrophy	sensory predominant neuropathy	PE, IVIG (ineffective)			48 months
P8		43/M	Progressive ataxia, dysarthria	Cerebellar ataxia	MRI brain: moderate cerebellar atrophy	NA	NA			3 months
P9		42/F	Gait abnormality, dysarthria, incoordination	Spinocerebellar syndrome	MRI brain: cerebellar atrophy; MRI cord: normal	EMG: normal	CS, PE, IVIG (ineffective)			40 months
P10		43/M	Ataxia, dysarthria, severe vertigo	Spinocerebellar syndrome	MRI brain: cerebellar atrophy	NA	NA			NA
P11	Liu et al./2021	47/M	Paresthesias, gait disturbance, muscle twitching	Cerebellar ataxia, Myeloneuropathy	MRI brain: cerebellar atrophy	Neurogenic change	IVIG (partially improved); CS, MMF (stable)		–	110 months
P12	Vilaseca et al./ 2023	73/M	Gait disturbance, dysesthesias in the lower limbs, dysarthria	Cerebellar ataxia	MRI brain, mild cerebellar atrophy	NA	None (stable)	None	Lumbar spinal stenosis, vascular leukoencephalopathy	30 months
P13		10/F	Gait disturbance, limb ataxia with predominant right dysmetria, intentional tremor, nausea, and vomiting	Cerebellar ataxia	MRI brain, mild cerebellar vermis atrophy; MRI cord: normal	NA	IVIG (slight improvement), RTX (slight improvement)	None	Generalized febrile seizures	36 months
P14	our patient/2024	55/F	Gait disturbance, limb numbness, pyramidal tract lesion	SCD mimic	MRI brain: normal; MRI cord: T2 signal in posterior columns	sensory neuropathy and absent SEP	Mecobalamin (ineffective)	None	None	72 months

CS, corticosteroids; CTX, cyclophosphamide; IVIG, intravenous immunoglobulin; LETM, longitudinally extensive transverse myelitis; MMF, mycophenolate mofetil; NA, not available; PE, plasma exchange; RTX, rituximab; SCD, subacute combined degeneration of spinal cord.

AP3B2 expression in dorsal root ganglia theoretically predisposes to anti-AP3B2 antibody-mediated sensory neuronal injury. SN encompasses a spectrum of neurological disorders sharing overlapping pathogenesis, clinical manifestations, and imaging features. Clinically, SN is characterized by an asymmetric and non-length-dependent sensory impairment at onset, manifesting as hypoesthesia or anesthesia, sensory ataxia, diminished tendon reflexes, and T2-hyperintense signals in the posterior spinal columns on MRI (8). While prior literature classifies AP3B2 antibody-associated sensory disturbances as sensory neuropathy (4), we propose that cases presenting solely with sensory deficits (e.g., limb numbness) should be more appropriately classified as AP3B2 antibody-associated SN to reflect precise clinicopathological correlation.

The heterotetrametric AP3 protein, composed of β, δ, μ, and σ subunits, serves as a crucial adaptor complex in vesicle biogenesis (5). The ubiquitously expressed AP3A subtype directs protein trafficking from endosomes to lysosomes in various cell types. In contrast, the neuron-specific AP3B2 isoform is localized to dendrites and axons and mediates targeted transport of proteins from endosomes to synaptic vesicles. Loss of AP3 function results in aberrant expression of neurotransmitters and ion transporters in synaptic vesicles, thereby impairing synaptic transmission. Studies suggest that AP3 not only regulates protein targeting to lysosomes and melanosomes but may also be implicated in the pathogenesis of psychiatric disorders and epileptic encephalopathies (9, 10). However, since AP3B2 is an intracellular antigen lacking transmembrane domains and is not exposed on the cell surface, antibodies against AP3B2 cannot bind to it. Thus, AP3B2 itself is unlikely to be a pathogenic autoantigen; rather, AP3B2 autoantibodies may serve as biomarkers for associated neurological disorders. These antibodies likely exert their effects via CD8^+^ T cell-mediated cytotoxic mechanisms. Similarly, in paraneoplastic SN associated with anti-Hu antibodies and cerebellar ataxia linked to anti-Yo antibodies, infiltration of CD8^+^ T cells has been observed in sensory neurons and the cerebellum, respectively (11, 12). This phenomenon is characteristic of all autoimmune disorders targeting intracellular antigens that are named after their corresponding antibodies. Although AP3B2 antibodies react with neuroectodermal tumor cell lines (5), they have not been classified as autoimmune disorders associated with paraneoplastic syndromes to date. Among reported AP3B2 antibody-positive patients, only two cases with tumors have been identified (P3 and P6 in **Table 1**), yet no temporal correlation was observed (4). We need further studies to explore the potential pathogenesis of AP3B2 antibody-associated autoimmune disorders.

Among all documented cases, merely two patients demonstrated mild responses to immunotherapy (6, 7). Regrettably, this SCD-mimic patient declined plasma exchange and other immunotherapies, precluding evaluation of treatment efficacy.

In conclusion, we report the first case of an SCD-mimic phenotype associated with anti-AP3B2 antibodies, thereby expanding the clinical spectrum of AP3B2 antibody-related disorders. Moving forward, clinicians should consider screening for anti-AP3B2 antibodies in patients presenting with SCD-like manifestations who exhibit poor responsiveness to vitamin B12 therapy.

## Data Availability

The original contributions presented in the study are included in the article/supplementary material. Further inquiries can be directed to the corresponding authors.
